# Determinants of Achilles tendon thickness and their influence on knee function and foot alignment in knee osteoarthritis

**DOI:** 10.1038/s41598-024-67932-8

**Published:** 2024-07-23

**Authors:** Shaikh Nabi Bukhsh Nazir, Basit Ansari

**Affiliations:** Department of Health, Physical Education, and Sports Sciences, University of Karachi, Karachi, Pakistan

**Keywords:** Knee joint, Osteoarthritis, Achilles tendon rupture, Tendon elongation, Rehabilitation, Osteoarthritis, Risk factors

## Abstract

Knee osteoarthritis (OA) significantly impacts the quality of life of individuals globally. However, the interconnections between Achilles tendon thickness, knee symptoms/functions, and foot alignment remain understudied in knee OA patients. This study determines the relationships between Achilles tendon thickness (ATT), knee symptoms/functions, and foot alignment in knee OA patients, considering their interconnected biomechanical nature. In a cross-sectional analysis involving 122 knee OA patients, Knee injury and Osteoarthritis Outcome Score (KOOS) assessed knee function and symptoms. Forefoot, midfoot, and rearfoot alignment were measured using hallux valgus angle, navicular/foot ratio, and rearfoot angle. The navicular/foot ratio represented the ratio of navicular height to total foot length. ATT was measured using a digital calliper. Pearson correlations and stepwise multiple linear regression models were employed to explore relationships and determinants. Out of 122 participants, 88 (72.1%) were females. ATT correlated significantly with ankle range of motion, forefoot alignment, and midfoot alignment. In stepwise multivariable regression, ankle range of motion, navicular/foot ratio, and age were significantly associated with ATT (adjusted R^2^ = 0.44). Similarly, KOOS-Symptoms scores were linked to the OA severity, navicular/foot ratio, ankle range of motion, gastrocnemius strength, and age (adjusted R^2^ = 0.22). KOOS-Function scores were significantly associated with knee OA severity, gastrocnemius strength, ankle range of motion, and age (adjusted R^2^ = 0.19). Midfoot alignment was significantly associated with ATT and knee symptoms in patients with Knee OA. This suggests potential benefits of interventions targeting both Achilles tendon properties and foot alignment for improved knee OA outcomes.

## Introduction

Knee osteoarthritis (OA) is a degenerative joint condition that has a profound impact on the quality of life and functional abilities of individuals worldwide^1,2^. It is characterized by edema and a reduction in joint cartilage, affecting the patellofemoral, medial, and lateral tibiofemoral compartments, potentially leading to joint deformities and impaired functioning^3^. Knee OA most frequently occurs in the medial compartment of the tibiofemoral joint, giving rise to kinetic and kinematic alterations in the lower limbs, as well as causing pain and decreased mobility^4^.

Patients with medial tibiofemoral Knee OA exhibit several kinematic alterations during their gait cycle. These include subtalar pronation, a more everted rearfoot upon initial contact, and the presence of pes planus, all of which are linked to increased pain and cartilage degradation in the knee joint and heel supination^5–8^. Additionally, individuals with Knee OA and varus alignment often display a greater rearfoot valgus^9^.

The multifactorial nature of knee OA involves intricate interactions between various anatomical structures and biomechanical factors within the lower extremities^10^. While knee OA research has traditionally focused primarily on the knee joint itself, recent studies increasingly emphasize the importance of considering the interconnectedness of the entire kinetic chain, from the Achilles tendon to foot alignment^11^. An understanding of these relationships can offer valuable insights into the etiology, progression, and management of knee OA.

The Achilles tendon, a critical component of the lower limb, serves as a vital link between the calf muscles and the calcaneus. Its role in lower limb biomechanics extends beyond the ankle joint, influencing the function and alignment of adjacent joints^12^. Knee symptoms and functional limitations, frequently experienced by individuals with knee OA, contribute to altered gait patterns and loading mechanics^13^. Such alterations may, in turn, affect foot alignment^13^. Despite the potential implications of these interrelationships, limited research has systematically explored the associations between Achilles tendon thickness (ATT), knee symptoms or functions, and foot alignment in knee OA patients.

The study aimed to investigate the relationship between ATT, knee symptoms or functions, and foot alignment in individuals with Knee OA, considering their interconnected biomechanical characteristics. It is hypothesized that midfoot alignment, often linked to flatfoot, may have a more significant association with ATT, knee symptoms, and function. By exploring the complex interaction of Achilles tendon properties, knee symptoms or functions, and foot alignment, this research contributes to our comprehensive understanding of the holistic biomechanical factors that affect knee OA. It also presents opportunities to enhance patient care.

## Methodology

The cross-sectional study was conducted at the Baqai Institute of Physical Therapy and Rehabilitation Medicine, Baqai Medical University, from February 2023 to August 2023. Approval for the study (BMU-EC/2-2023/02) was obtained from the Institutional Review and Ethics Board of Baqai Medical University. A total of 122 patients with Knee OA (34 males and 88 females; mean age of 53.54 ± 8.80 years) were included in the study. A non-probability purposive sampling technique was employed for participant selection.

Patients with knee OA were screened and referred by a physiatrist, orthopedic surgeon, and general physician. Inclusion criteria included patients aged over 40 years with a confirmed diagnosis based on the American College of Rheumatology and Kellgren Lawrence radiological criteria (grades 2 to 4). Exclusion criteria included posttraumatic arthritis, intraarticular injections within the past 3 months, rheumatoid arthritis, tenderness over the Achilles tendon during clinical examination, constitutional or nonspecific symptoms, a body mass index exceeding 45 kg/m^2^, knee or Achilles tendon ulcerations, and severe lower limb joint deformities. Participants provided informed consent before their involvement in the study.

## Data collection

The dorsiflexion range of ankle movement was measured using the knee-to-wall lunge method, assessing the distance in centimeters between the big toe and the wall while maintaining ground contact^14,15^.

The strength of the gastrocnemius muscle was assessed using the manual muscle test method. To perform this test, the subject stood on the limb to be examined with the knee fully extended, targeting the gastrocnemius muscles. They were instructed to lift their heel from the floor, performing consecutive movements throughout the complete range of plantar flexion. The physical therapist provided a visual demonstration of the correct heel rise technique to the patient, with the accompanying verbal instructions: "Stand on one leg, rise up on your tiptoes, and then lower your heel. Repeat this movement as many times as you can." The grading system ranged from 3 to 5. Grade 5 was assigned when the subject completed 25 heel raises from the floor, covering the entire range of plantar flexion motion. Grade 4 required completing 11 to 24 heel raises, but with increased difficulty in executing the movement as they progressed. Grade 3 corresponded to completing 1 to 10 heel raises. Lastly, for grade 2, the patient was unable to complete even a single heel rise, though they could raise the heel slightly off the floor^16^.

## Achilles tendon thickness

The measurement of Achilles tendon thickness (ATT) was taken using a digital calliper (Digital Calipers by Adoric), which is a simple device displaying measurements in inches and millimeters^12^. The measurement was taken at the point where the ankle joint joins the medial malleolus, around 5 cm from the point where it attaches to the calcaneus, at a 90° angle^17,18^.

The Knee injury and Osteoarthritis Outcome Score (KOOS) is a validated patient-centered outcome questionnaire designed to assess the level of difficulty individuals experience when performing activities due to knee problems. In this research, the Urdu language version of KOOS was utilized. Two specific components of KOOS were used: KOOS-symptoms (with an ICC of 0.95) and activities of daily living (with an ICC of 0.99)^19^. For each sub-domain, scores ranging from 0 (indicating extreme problems) to 100 (indicating smooth functioning) were obtained separately.

## Foot alignment

The alignment of the affected foot was evaluated while the individual stood with 50% of their body weight distributed evenly on both feet^7^.

To assess the forefoot alignment, a goniometer was employed to measure the angle formed by the intersection of the first metatarsal axis and the first proximal phalanx of the forefoot^20^. This measurement is known as the hallux valgus angle, and it serves as an indicator of forefoot alignment^13^.

The midfoot alignment was assessed using the navicular/foot ratio, which is calculated as the ratio of the navicular height to the total foot length. Navicular height was determined by measuring from the ground to the navicular tuberosity, while the total foot length was measured from the calcaneus to the second toe. This method was chosen to account for variations in arch structures due to differences in foot length. Previous research has demonstrated the reliability of the navicular/foot ratio as a midfoot index ^21^.

For the evaluation of rearfoot alignment, we utilized the leg heel alignment (LHA) angle. This angle is defined as the measurement between the midline of the distal leg and the axis of the calcaneus^22,23^. A positive value indicates inversion, whereas a negative value signifies eversion.

Descriptive statistics were used to summarize participant demographics and characteristics. Pearson correlation coefficients were calculated to assess the relationships between ATT, knee symptoms or functions (KOOS subscales), and foot alignment. Independent variables were identified by using a multivariable linear stepwise regression model with all factors being initially included. Afterwards, the factors were excluded step by step until only those being significant remained. *P* < 0.05 was regarded as significant, and all statistical tests were performed using SPSS 23.0.

### Ethics approval and consent to participate

Ethical approval was obtained from Institutional Review and Ethics Board of Baqai Medical University (BMU-EC/2-2023/02). All study participants gave full informed written consent to participate in the study. All methods were carried out in accordance with relevant guidelines and regulations.

## Results

The study involved 122 participants, and notable physical characteristics included Achilles tendon thickness (mean 22.43 ± 2.35), foot alignment parameters (hallux valgus angle mean 23.86 ± 2.52, Navicular/foot ratio mean 0.22 ± 0.05, leg heel alignment angle mean -4.02 ± 2.91), and ankle range of motion (mean 25.18 ± 3.00). In terms of the severity of radiographic Knee OA, 38 participants (31.14%) were classified as having grade 2, followed by 58 participants (47.54%) at grade 3, and 26 participants (21.32%) at grade 4 knee OA. Gastrocnemius muscle strength was predominantly graded as 3 (72.1%) and 4 (27.9%). (Referring to Table 1).

**Table 1:** The characteristics of study participant (n = 122).

Characteristics	Mean ± SD or n (%)
Achilles tendon thickness	22.43 ± 2.35
Foot alignment	
Hallux valgus angle	23.86 ± 2.52
Navicular/foot ratio	0.22 ± 0.05
Leg heel alignment angle	− 4.02 ± 2.91
KOOS-pain	67.03 ± 22.37
KOOS-function	60.80 ± 22.17
Ankle ROM	25.18 ± 3.00
Gender	
Male	34 (27.9%)
Female	88 (72.1%)
Radiographic OA severities	
Grade 2	38 (31.14%)
Grade 3	58 (47.54%)
Grade 4	26 (21.32%)
Gastrocnemius muscle strength	
Grade 3	88 (72.1%)
Grade 4	34 (27.9%)

Table 2 shows the Pearson correlations between several variables, including ATT, Hallux Valgus Angle, Navicular/Foot Ratio, LHA Angle (Leg Heel Alignment Angle), gastrocnemius strength, and ankle Range of Motion (ROM). Hallux Valgus Angle (r = 0.233*, *P* < 0.01), Navicular/Foot Ratio (r = 0.244*, *P* < 0.01), and Ankle Range of Motion (r = 0.667, *P* < 0.01) exhibited significant correlations with ATT. Furthermore, Hallux Valgus (r = 0.191*, *P* < 0.05) demonstrated a significant correlation with ankle Range of Motion.

**Table 2:** Correlation among ATT, Hallux Valgus Angle, Navicular/foot ratio, LHA angle, gastrocnemius strength and ankle ROM.

Variables	ATT	Hallux valgus angle	Navicular/foot ratio	LHA angle	Gastrocnemius strength	Ankle ROM
ATT	1					
Hallux valgus angle	0.233**	1				
Navicular/foot ratio	0.244**	0.118	1			
LHA angle	0.121	0.031	− 0.096	1		
Gastrocnemius strength	0.091	0.098	− 0.153	0.026	1	
Ankle ROM	0.667**	0.191*	0.111	0.049	0.007	1

*Correlation is significant at the 0.05 level (2-tailed); **Correlation is significant at the 0.01 level (2-tailed).

In Table 3, the factors that determine ATT are presented through a stepwise multivariable linear regression analysis. Only three were identified as predictors of ATT from the eight remaining characteristics. The R-squared values indicated the proportion of variance in ATT scores explained by the independent variables, with significant percentages ranging from 44% to 44.8%. Ankle range of motion (ROM), navicular/foot ratio, and age were all significantly associated with ATT.

**Table 3:** Associations between ATT and foot alignments and other variables, using stepwise multiple linear regression analyses.

Variables	*Β*	95% confidence interval [CI]		R^2^ (%) (variance)	*p* value
		Lower bound	Upper bound		
Ankle ROM	0.654	0.410	0.613	44	0.000
Navicular/foot ratio	0.172	1.869	13.249	44.6	0.011
Age	− 0.168	− 0.079	− 0.011	44.8	0.011

*p* value was calculated by using multiple linear stepwise regression with age, radiographic OA severities, navicular/foot ratio, body mass index, hallux valgus angle, leg heel alignment, gastrocnemius strength, and ankle range of motion being initially included into the model.

Table 4 shows the outcomes of a stepwise multiple linear regression analysis investigating the relationship between KOOS symptoms and various factors. The results indicate that Radiographic OA severity has the highest Β at 0.37, followed by Navicular/foot ratio at 0.30, Ankle ROM at 0.27, Gastrocnemius strength at 0.21, and age at 0.20. These findings suggest that factors such as radiographic OA severity, foot alignment (as indicated by the navicular/foot ratio), ankle range of motion, gastrocnemius strength, and age significantly influence the KOOS symptoms.

**Table 4:** Associations between KOOS-Symptoms and foot alignments and other variables, using stepwise multiple linear regression analyses.

Variables	*Β*	95% confidence interval [CI]		R^2^ (%) (variance)	*p* value
		Lower bound	Upper bound		
Radiographic OA severities	0.37	6.491	16.503	9.1	0.000
Navicular/foot ratio	0.3	2.2	14.2	13.4	0.000
Ankle ROM	0.27	0.85	3.207	15.7	0.002
Gastrocnemius strength	0.21	1.122	7.798	19.4	0.013
Age	0.2	0.922	0.103	22.7	0.015

*p* value was calculated by using multiple linear stepwise regression with age, radiographic OA severities, navicular/foot ratio, body mass index, hallux valgus angle, leg heel alignment, gastrocnemius strength, and ankle range of motion being initially included into the model.

Table 5 presents a stepwise multiple linear regression analysis examining the association between KOOS-Function and various factors. Only four were identified as independent risk factors for KOOS- Function from the eight remaining characteristics. The R-squared values indicated the proportion of variance in KOOS-Function scores explained by the independent variables, with significant percentages ranging from 9.4 to 19.4%. Radiographic knee OA, gastrocnemius strength, ankle range of motion, and age were all significantly associated with KOOS-Function scores.

**Table 5:** Associations between KOOS-Function and foot alignments and other variables, using stepwise multiple linear regression analyses.

Variables	*Β*	95% confidence interval [CI]		R^2^ (%) (variance)	*p* value
		Lower bound	Upper bound		
Radiographic OA severities	0.34	5.408	15.54	9.4	0.001
Gastrocnemius strength	0.268	2.205	8.961	13.4	0.002
Ankle ROM	0.2	0.289	2.674	15.7	0.021
Age	− 0.168	− 0.851	− 0.023	19.4	0.039

*p* value was calculated by using multiple linear stepwise regression with age, radiographic OA severities, navicular/foot ratio, body mass index, hallux valgus angle, leg heel alignment, gastrocnemius strength, and ankle range of motion being initially included into the model.

## Discussion

This study aimed to investigate the potential relationship between Achilles tendon thickness (ATT), knee symptoms or functions, and foot alignment among patients diagnosed with Knee OA. The results of this study provide valuable insights into the intricate interplay of lower limb structures in individuals with knee OA. The navicular-to-foot ratio, as a measure of midfoot alignment, is significantly associated with ATT and KOOS-Symptoms. Meanwhile, age and ankle range of motion are significantly associated with KOOS-Function, ATT, and KOOS-Symptoms. Moreover, the severity of radiographic knee OA and gastrocnemius muscle strength are significantly associated with KOOS-Symptoms and Function.

Age influences the thickness of the Achilles tendon in individuals with knee OA. The study indicates that structural changes in the tendon due to aging, such as increased stiffness and reduced elasticity, play a significant role in the variations in ATT observed in individuals affected by knee OA^11,12^. This finding is consistent with other research that highlights age as a critical factor influencing tendon morphology^24^. It also sheds light on the complex relationship between age-related structural changes and ATT in the context of knee OA, emphasizing the impact of aging on tendon biomechanics.

The association between ATT, navicular/foot ratio, and ankle range of motion provides valuable insights into the biomechanics of the lower limb and possible implications for tendon adaptations^25^. Foot posture, which is a crucial factor affecting leg muscle mass, can lead to changes in muscle properties such as tone and stiffness^26^. Individuals with medial tibiofemoral Knee OA often exhibit a more pronated foot posture and calcaneus valgus angle^27^. These factors are linked to alterations in varus or valgus alignment, knee adduction moment, and limb/trunk alignment, ultimately increasing the load on the medial knee and reducing joint space^28^. The negative correlation between the width of the medial tibiofemoral joint and knee adduction moment in individuals with knee OA is attributed to increased co-contraction around the knee and ankle involving the Achilles tendon and gastrocnemius muscle^29^. This clarifies the relationship between foot posture, ankle range of motion, and Achilles tendon thickness, providing valuable insights into the biomechanical aspects of knee OA^30,31^. Increased ATT in patients with knee OA suggests potential adaptations to altered biomechanics, serving as a protective mechanism by redistributing forces and minimizing discomfort in the affected knee. Changes in foot alignment also significantly impact tendon thickness, highlighting the interconnected nature of lower limb biomechanics in knee OA^11^. This finding underscores the complex interplay between foot alignment, tendon adaptations, and biomechanical adjustments contributing to ATT in knee OA.

Our study has revealed a significant association between knee symptoms and foot alignment in the context of knee OA. Patients with more severe knee symptoms tend to exhibit specific foot malalignments, including a reduced navicular/foot ratio, often accompanied by a weakened gastrocnemius muscle and limited ankle range of motion. These findings suggest a two-way association between knee symptoms and foot alignment, indicating that knee symptoms may impact gait patterns and foot mechanics, leading to adjustments in foot alignment. Abnormal foot alignment could alter the load distribution on the knee joint, potentially exacerbating existing symptoms. Conversely, knee symptoms could affect gait patterns and foot mechanics, resulting in changes in foot alignment. Additionally, knee symptoms may cause individuals to adjust their gait to reduce medial knee joint load. A lateral sway of the trunk can effectively lower ipsilateral knee adduction moments, thereby minimizing pain and preventing further damage^33,34^. This compensatory mechanism aligns with findings from Fukaya's study, which observed that reduced ankle movement during walking can change how weight and pressure are distributed on the foot^33,34^. This adaptation may help in managing knee symptoms by altering foot mechanics and load distribution.

The present study has revealed a significant association between the navicular-to-foot ratio and KOOS-Symptoms. However, this association was not observed with the Hallux valgus angle and the Leg heel alignment angle. According to kinematic theory, foot adduction is known to cause simultaneous rearfoot eversion, hallux valgus, and navicular drop. Interestingly, our findings contradict a previous study involving Japanese individuals, where no such association was found among rearfoot eversion, hallux valgus, and navicular drop^35,36^.

A research study found that individuals with bilateral Hallux Valgus tend to shift their center of pressure away from the first metatarsophalangeal joint^37^. This reduces foot loading but leads to increased knee abductor moments^37^. Additionally, another study suggests that Hallux Valgus impairment is associated with increased ankle moments during gait, which may contribute to ankle osteoarthrosis^38^. Alizadeh et al. observed that people with Hallux Valgus tend to have a greater foot angle during mid-stance and terminal stance, which may be a way to alleviate pain and decrease pressure on the hallux^39^.

Studies have shown that differences in navicular height may play a role in hip and medial compartment knee OA^40,41^. This underscores the significance of foot characteristics in various forms of lower limb osteoarthritis. As navicular height decreases, it can affect the kinematic chain by increasing knee internal rotation, foot adduction, and tibia eversion^42^. Our research indicates that specific foot alignment measures, such as lower navicular height, could impact knee symptoms and moments, which may lead to increased pain and an increased risk of knee OA. Understanding the interplay between foot alignment measures and knee symptoms can provide valuable insights into the complex biomechanics of lower limb osteoarthritis. This underscores the need for comprehensive treatment approaches that address the entire kinetic chain to enhance patient outcomes.

Our study revealed that knee functions and foot parameters have a significant correlation. Patients experiencing impaired knee functions demonstrated a notable inclination towards decreased gastrocnemius muscle strength and limited range of motion. These results are consistent with prior research, which showed weakened plantarflexor muscles and reduced ankle range of motion^43–45^. The findings highlight the intricate interdependency of lower limb biomechanics in individuals with knee OA. It is possible that compromised knee functions lead to altered gait patterns, which, in turn, affect foot parameters^12,46^. Therefore, clinicians and researchers must consider these complex interactions when developing treatment strategies for patients with knee OA. Targeted interventions that aim to improve both knee functions and foot alignment may offer a more comprehensive and effective approach to managing the condition. These interventions could consist of stretching exercises to enhance ankle range of motion, strengthening the gastrocnemius muscle for improved stability, implementing orthotic interventions to correct foot alignment problems such as hallux valgus, and providing gait training to optimize lower limb biomechanics. Furthermore, weight management programs and pain management techniques can complement these interventions, providing a holistic and multifaceted approach to managing knee OA, ultimately enhancing patient outcomes and quality of life.

When interpreting the implications of our findings, it is important to acknowledge certain limitations of the study. The cross-sectional design precludes establishing causal relationships between Achilles tendon thickness (ATT), knee symptoms or functions, and foot alignment. Future studies should consider a longitudinal study design to better understand the evolution of these factors and their mutual influences over time. Additionally, our study only focused on patients with knee OA, which may limit the generalizability of our findings to wider populations. Including control groups without knee OA would be valuable to address this limitation and provide comparative insights. Radiographic assessment of angles, such as supination and pronation, was not included in this study, which could yield different perspectives on foot alignment and its relationship to knee symptoms. Lastly, the data collection by a single observer is a potential limitation that future studies might address by involving multiple observers for data validation.

## Conclusion

The study's findings provide insights into the complex interactions between ATT, knee symptoms or functions, and foot alignment in individuals with knee OA. Notably, the navicular/foot ratio, indicative of midfoot alignment, shows a significant association with both ATT and knee symptoms in patients with medial knee OA. These results underscore the need for further research to understand the underlying mechanisms of these relationships and to develop therapeutic approaches that concurrently address Achilles tendon characteristics and foot alignment, potentially enhancing treatment outcomes for knee OA patients.

## Data Availability

Data included in the current study are not publicly available to ensure confidentiality of the patients but are available from the corresponding author on reasonable request.

## Abbreviations

K/L: Kellgren lawrence
Knee OA: Knee osteoarthritis
KOOS: Knee injury and Osteoarthritis Outcome Score
